# The Spanish Fabry women study: a retrospective observational study describing the phenotype of females with *GLA* variants

**DOI:** 10.1186/s13023-022-02599-w

**Published:** 2023-01-09

**Authors:** Rosario Sánchez, Tomás Ripoll-Vera, Manuel López-Mendoza, Joaquín de Juan-Ribera, Juan Ramón Gimeno, Álvaro Hermida, María Aurora Ruz-Zafra, José Vicente Torregrosa, Antonia Mora, José Manuel García-Pinilla, Elena Fortuny, Ana Aguinaga-Barrilero, Roser Torra

**Affiliations:** 1 Multidisciplinary Unit for Low Prevalence Diseases, Hospital General Universitario de Alicante, Alicante, Spain; 2 Hospital Universitario Son Llàtzer & IdISBa, Palma de Mallorca, Spain; 3 Hospital Universitario Virgen del Rocío, Seville, Spain; 4 Hospital General Universitario de Elda, Elda, Spain; 5 Hospital Universitario Virgen de La Arrixaca, Murcia, Spain; 6 Complexo Hospitalario Universitario de Santiago de Compostela, Santiago de Compostela, Spain; 7 Hospital Serranía de Ronda, Ronda, Spain; 8 Hospital Clinic, Barcelona, Spain; 9 Hospital General Universitario de Elche, Elche, Spain; 10 Hospital Universitario Virgen de La Victoria, IBIMA, Málaga, Ciber-Cardiovascular, Instituto de Salud Carlos III, Departamento de Medicina y Dermatología, Universidad de Málaga, Málaga, Spain; 11 Hospital Universitario Son Espases & IdISBa, Palma, Spain; 12 Amicus Therapeutics, SLU, Madrid, Spain; 13 Inherited Kidney Diseases, Nephrology Department, Fundació Puigvert, Institut d’Investigació Biomèdica Sant Pau (IIB-SANT PAU), Barcelona, Spain

**Keywords:** Fabry disease, X-linked disorder, GLA variants, Females, Organ involvement

## Abstract

**Background:**

Fabry disease (FD) is an X-linked condition caused by variants in the *GLA* gene. Since females have two X chromosomes, they were historically thought to be carriers. Although increased knowledge has shown that females often develop the disease, data from Spain and other countries reported that females were undertreated. The aim of this study was to provide a wider and more recent description of the disease characteristics and associated management of females with a *GLA* variant in a Spanish cohort.

**Results:**

Ninety-seven females from 12 hospitals were included in this retrospective study. Mean age was 50.1 ± 17.2 years. Median follow-up time from *GLA* variant identification was 36.1 months, and most (70.1%) were identified through family screening. Variants associated with classic/non-classic phenotypes were similarly distributed (40.2%/53.6%). Missense variants were the most prevalent (n = 84, 86.6%). In the overall group, 70.4% had major organ involvement (i.e., cardiac, renal, cerebrovascular, peripheral nervous system or gastrointestinal), and 47.3% also had typical Fabry signs (angiokeratoma, cornea verticillata or increased plasma lyso-Gb3). Cardiac involvement was the most prevalent (49.5%) and the main reason for treatment initiation. A total of 33 (34%) patients received disease-specific therapy, 55% of whom were diagnosed by family screening. Females carrying variants associated with a classic phenotype had higher frequencies of clinical manifestations (92.3%) and were predominant in the treated subgroup (69.7%). Despite this, there were 34 untreated females (56.7% of total untreated), with both phenotypes represented, who had major organ involvement, with 27 of cardiac, renal or cerebrovascular nature. Age or comorbidities in this subgroup were comparable to the treated subgroup (*P* = 0.8 and *P* = 0.8, respectively).

**Conclusions:**

Efforts have been made in recent years to diagnose and treat timely Fabry females in Spain. A high percentage of females with pathogenic variants, regardless of their associated phenotype, will likely develop disease. A proportion of females with severe disease in this cohort received specific treatment. Still a significant number of females, even with same profile as the treated ones, who may be eligible for treatment according to European recommendations, remained untreated. Reasons for this merit further investigation.

**Supplementary Information:**

The online version contains supplementary material available at 10.1186/s13023-022-02599-w.

## Introduction

Fabry disease (FD) is an X-linked inherited lysosomal disease associated with mutations of the gene (*GLA*) encoding the enzyme α-galactosidase A (α-Gal A). The result is a total or partial loss of its function, leading to lysosomal accumulation of complex glycosphingolipids, primarily globotriaosylceramide (Gb3) and its metabolites in plasma and urine, as well as in a variety of cell types, particularly in the kidney, heart, and nervous system. This results in cellular dysfunction, inflammation, and progressive organ damage [1–4].

The annual incidence of FD in new born males has been estimated to be 1:40,000–1:60,000 [5, 6]. Recent newborn screening studies in Italy, Taiwan, Austria, Spain, and the U.S., which screened more than 500,000 male and female newborns, found the incidence of FD mutations to be between 1:2445 to 1:8454, more than ten times higher than previous estimates for classic patients [7–11]. When looking at only male newborns within these studies, the incidence of FD mutations is as high as 1:1316–1:7575 [8, 12, 13]. However, this must be taken with caution, since many of these studies include variants of unknown significance (VUS).

Although there is consensus that FD has two broad phenotypes, classic (early-onset) and non-classic (later onset), FD comprises a clinical continuum with a wide spectrum of clinical phenotypes [1], especially in females. Symptoms vary in type and severity though they tend to increase in severity with age leading to poorer quality of life and shorter lifespan compared to the general population [1, 14, 15]. As a multisystem disease with non-specific symptoms which may overlap with other diseases, there is often a delay between the onset of symptoms and diagnosis of up to 10–15 years or even longer in females [16]. Avoiding delayed diagnosis is crucial to increase the quality and expectancy of life of the patients who need a Fabry-specific treatment. Family screening is encouraged as a tool to increase the diagnostic rate of FD [17].

Though this disease is X-linked, both males and females are affected. Clinical presentation in female patients may be more variable due to random X-chromosome inactivation, and ranges from asymptomatic to equally severe as classically affected male patients [18–20].

The difference in clinical expression between males and females in FD is reflected in the European recommendations for specific treatment initiation [21–23], with recommendations separately defined for males and females. For females, irrespective of whether the mutation is classified as classic or non-classic, demonstrating the presence of signs or symptoms of Fabry disease (ruling out other causes) is always required [20, 21, 24].

In Spain, as well as in other countries where this was studied [25, 26], it was reported several years ago that females were undertreated according to published recommendations at the time (Spanish Consensus Recommendations from 2011) [27].

In view of the above, there is an ongoing need for further characterisation of FD in females, and for a better understanding of how they are being managed. The aim of this study was to provide a more recent and wider description of the disease characteristics and associated management of females with a *GLA* variant in a Spanish cohort.

## Results

### Patient characteristics

Between November 2020 to April 2021, a total of 97 females with a variant in the *GLA* gene were included in the study. Baseline characteristics of the entire cohort, by type of variant associated phenotype based on the literature, and by treatment status (treated vs untreated) are described in Table 1. Briefly, median age (range) was 50 (16–84) years, the mean (standard deviation, SD) follow-up time since *GLA* variant identification until last outpatient visit or death was 52.9 (47.9) months (median 36.1 [3–226]), and most females (70.1%) were identified through family screening. Thirty-nine (40.2%) females had a classic variant, 52 (53.6%) a non-classic variant, and 6 (6.2%) had a variant of uncertain significance (VUS). A total of 53 (54.6%) patients had at least one comorbidity, mainly arterial hypertension (25.8%), and diabetes mellitus (7.2%). Regarding treatment status, 15 (51.7%) patients out of the 29 diagnosed by clinical suspicion, and 18 (26.5%) out of 68 who were diagnosed by family screening had received specific therapy for FD. Thus, 33 (34.0%) patients were treated in total, and most carried a classic gene variant, significantly different from the untreated subgroup where most females presented with a non-classic variant (*P* < 0.001). There was no difference in age between treated and untreated subgroups. On the other hand, there were significant differences in the presence of any comorbidity based on treatment status (*P* = 0.032). Most treated females had some type of comorbidity (67%), while the untreated subgroup had similar rates of females with (47%) and without (53%) comorbidities. Plasma lyso-Gb3 was more frequently elevated in patients with classic variants (*P* < 0.001) and also in treated patients (*P* < 0.001). However, there were no statistically significant differences in peripheral blood α-Gal A activity between treated and untreated patients or according to phenotype. Of the 33 patients who had received specific therapy for FD, 28 (84.8%) started treatment upon *GLA* variant identification and 5 (15.2%) during the follow-up period. Two treated patients had VUS and were excluded from subsequent analysis, together with the other 4 untreated females with VUS.

**Table 1:** Demographic and baseline disease characteristics

Patient characteristics	Overall N = 97	Type of variant regarding associated phenotype			Treatment status		
		Classic N = 39	Non-classic N = 52	*P* value	Treated N = 33	Untreated N = 64	*P* value
Age at study inclusion, years				0.683^b^			0.127^b^
Median (range)	50.0 (16–84)	51.0 (16–81)	50.5 (19–84)		52.0 (16–79)	45.5 (16–84)	
Mean (SD)	50.1 (17.2)	49.3 (17.2)	50.8 (17.6)		53.8 (14.8)	48.2 (18.1)	
Identification of the mutation, n (%)				0.071^c^			**0.016**^**c**^
Family screening	68 (70.1)	24 (61.5)	41 (78.9)		18 (54.6)	50 (78.1)	
Clinical signs/symptoms	29 (29.9)	15 (38.5)	11 (21.2)		15 (45.5)	14 (21.9)	
Type of variant regarding associated phenotype, n (%)				**< 0.001**^d^			< 0.001^d^
Classic	39 (40.2)	39 (100.0)	0 (0.0)		23 (69.7)	16 (25.0)	
Non-classic	52 (53.6)	0 (0.0)	52 (100.0)		8 (24.2)	44 (68.8)	
VUS	6 (6.2)	0 (0.0)	0 (0.0)		2 (6.1)	4 (6.3)	
Plasma Lyso-Gb3 levels, n (%)	48 (49.5)	12 (30.8)	33 (63.5)		14 (42.4)	34 (53.1)	
Median (range), ng/mL	2.4 (1–11)	6.5 (3–11)	1.8 (1–11)	**< 0.001**^e^	6.5 (1–11)	1.8 (1–5)	**< 0.001**^e^
Mean (SD), ng/mL	3.4 (2.6)	6.4 (2.2)	2.5 (1.8)		6.1 (3.0)	2.3 (1.2)	
Elevated, n (%)	24 (50.0)	12 (100.0)	12 (36.4)	**< 0.001**^c^	13 (92.9)	11 (32.4)	**< 0.001**^c^
α -Gal A activity, n (%)	29 (29.9)	10 (25.6)	16 (30.8)		14 (42.4)	15 (23.4)	
Decreased^a^, n (%)	15 (51.7)	6 (60.0)	9 (56.3)	1.000^d^	9 (64.3)	6 (42.9)	0.256^c^
Any comorbidity, n (%) Most frequent comorbidities, n (%)	53 (54.6)	19 (48.7)	30 (57.7)	0.395^c^	23 (69.7)	30 (46.9)	**0.032**^**c**^
Arterial hypertension	25 (25.8)	11 (28.2)	13 (25.0)		14 (42.2)	11 (17.2)	
Diabetes mellitus	7 (7.2)	3 (7.7)	4 (7.7)		1 (3.0)	6 (9.4)	
Obesity	6 (6.2)	1 (2.6)	4 (7.7)		3 (9.1)	3 (4.7)	
Fibromyalgia	3 (3.1)	2 (5.1)	1 (1.9)		2 (6.1)	1 (1.6)	
Genetic variant, n (%)				**< 0.001**^d^			**< 0.001**^d^
Nonsense	7 (7.2)	5 (12.8)	2 (3.9)		6 (18.2)	1 (1.6)	
Missense	84 (86.6)	28 (71.8)	50 (96.2)		23 (69.7)	61 (95.3)	
Deletion	1 (1.0)	1 (2.6)	0 (0.0)		1 (3.0)	0 (0.0)	
Frameshift	5 (5.2)	5 (12.8)	0 (0.0)		3 (9.1)	2 (3.1)	

For phenotype analysis females carrying VUS were excluded

α-Gal A, alpha-galactosidase A; Lyso-Gb3, plasma globotriaosylsphingosine; NA, not available; VUS, variant with unknown significance

^a^Increased or decreased levels were compared to the reference values of each site per their methodology; ^b^T-test; ^c^X^2^-square; ^d^Fisher's; ^e^Mann–Whitney

Statistically significant *p* values (*p* <0.05) are highlighted in bold

### *GLA* variant profile

Twenty-nine different variants in the *GLA* gene were identified among the 97 patients. Nineteen of these were missense variants (n = 84, 86.6%), 6 were nonsense variants (n = 7, 7.2%), 3 were insertions or deletions of nucleotides that cause a frameshift (n = 5, 5.2%) and 1 was a deletion (n = 1, 1.0%). The most prevalent mutations found were p.Ser238Asn (37.1%), p.Arg301Gln (10.3%) and p.Met187Arg (9.3%). A total of 21 (72.4%) detected variants were associated with a classic phenotype, 5 (17.2%) with a non-classic, and 3 (10.3%) were VUS (Table 2).

**Table 2:** Genetic variants

Genetic mutation	Variant	Associated phenotype	n (%)
p.Ala143Thr (c.427G>A)	Missense	VUS	2 (2.1)
p.Asp313Gly (c.938A>G)	Missense	Classic	4 (4.1)
p.Asp313Tyr/p.Gly411Asp (c.937G>T; 1232G>A)	Missense	Classic	1 (1.0)
p.Gly147Glu (c.440G>A)	Missense	Classic	1 (1.0)
p.Gly373Asp (c.1118G>A)	Missense	Classic	1 (1.0)
p.Gly411Asp (c.1232G>A)	Missense	Classic	1 (1.0)
p.Ile270Thr (c.809T>C)	Missense	Classic	1 (1.0)
p.Lys185* (c.553A>T)	Nonsense	Classic^a^	1 (1.0)
p.Met187Arg (c.560T>G)	Missense	Classic	9 (9.3)
p.Met187Thr (c.560T>C)	Missense	Classic	1 (1.0)
p.Met290Ile (c.870G>A o c.870G>C o c.870G>T)	Missense	Non-classic^a^	3 (3.1)
p.Met290Thr (c.869T>C)	Missense	Classic^a^	2 (2.1)
Other: c.9_19del	Frameshift	Classic^a^	3 (3.1)
Other: p.Gly163* (c.487 G>T)	Nonsense	Non-classic^a^	2 (2.1)
p.Gln119* (c.355C>T)	Nonsense	Classic	1 (1.0)
p.Gln250Pro (c.749A>C)	Missense	Classic	5 (5.2)
p.Arg118Cys (c.352C>T)	Missense	VUS	3 (3.1)
p.Arg227* (c.679C>T)	Nonsense	Classic	1 (1.0)
p.Arg301Gln (c.902G>A)	Missense	Non-classic	10 (10.3)
p.Arg363His (c.1088G>A)	Missense	Non-classic	1 (1.0)
p.Ser126Gly (c.376A>G)	Missense	VUS	1 (1.0)
p.Ser238Asn (c.713G>A)	Missense	Non-classic	36 (37.1)
p.Trp162Gly (c.484T>G)	Missense	Classic	1 (1.0)
p.Trp262* (c.785G>A o c.786G>A)	Nonsense	Classic	1 (1.0)
p.Trp81Ser (c.242G>C)	Missense	Classic	1 (1.0)
p.Trp95* (c.284G>A o c.285G>A)	Nonsense	Classic	1 (1.0)
p.358delGlu (c.1072_1074del)	Deletion	Classic	1 (1.0)
p.Ala368delinsFYfs*23 (c.1102delGinsTTATAC)	Frameshift	Classic^a^	1 (1.0)
p.Lys426Argfs*23 (c.1277_1278del)	Frameshift	Classic	1 (1.0)

VUS, variant of unknown significance

^a^Variants found to be associated with both phenotypes in the literature or not published. Phenotype was then assigned based on the investigator’s experience

### Clinical involvement

Overall clinical characteristics as well as differences between subgroups were analysed excluding cases with VUS, to avoid interference from controversial variants. The clinical characteristics of cases with VUS are described in Additional file 1: Table S1.

**Fig. 1:**
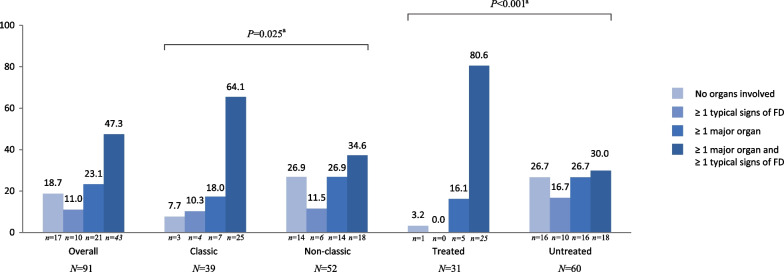
Distribution of major organ involvement and typical Fabry signs in this Fabry female cohort (females with VUS excluded). Major organ included cardiac, renal, PNS, cerebrovascular and GI involvement; typical signs of FD were angiokeratoma, cornea verticillata, and elevated Lyso-Gb3. ^a^X2 test. Abbreviations: FD, Fabry disease; GI, gastrointestinal; PNS, peripheral nervous system

Table 3 shows signs and symptoms of this cohort whether present at the time of diagnosis or at last outpatient visit. A total of 76 (83.5%) patients had clinical symptoms or signs. The most prevalent organ involvement was cardiac (49.5%), followed by ophthalmological (38.5%), peripheral nervous system (PNS) (35.2%) and renal (19.8%). Predominant signs and symptoms per organ system were left ventricular hypertrophy (LVH, 38.5%), cornea verticillata (29.7%), neuropathic pain (23.1%) and acroparesthesias (23.1%) (Table 3), with 70.4% of all patients having at least one major organ system involvement (i.e., cardiac, renal, cerebrovascular, PNS or gastrointestinal), and 47.3% having major organ involvement together with a typical Fabry sign (angiokeratoma, cornea verticillata or increased plasma lyso-Gb3) (Fig. 1). These proportions increased to 82.1% and 64.1% within the subgroup with a classic variant, and to 96.7% and 80.6% in the treated females, being a significant difference when compared to non-classic (61.5% and 34.6%; *P* = 0.025) and to untreated females (56.7% and 30.0%; *P* < 0.001), respectively. (Fig. 1).

**Table 3:** Female signs and symptoms at diagnosis or at last assessment (females with VUS excluded)

Type of signs or symptoms	Total N = 91	Type of variant regarding associated phenotype			Treatment status		
		Classic N = 39	Non-classic N = 52	*P* value	Treated N = 31	Untreated N = 60	*P *value
Any^a^	76 (83.5)	36 (92.3)	40 (76.9)	0.050^d^	30 (96.8)	46 (76.7)	**0.014**^**d**^
Peripheral nervous system	32 (35.2)	22 (56.4)	10 (19.2)	**< 0.001**^**d**^	15 (48.3)	17 (28.3)	0.058^d^
Neuropathic pain	21 (23.1)	18 (46.2)	3 (5.8)		8 (25.8)	13 (21.7)	
Acroparesthesia	21 (23.1)	16 (41.0)	5 (9.6)		13 (41.9)	8 (13.3)	
Heat/cold intolerance	6 (6.6)	6 (15.4)	0 (0.0)		5 (16.1)	1 (1.7)	
Sickness	8 (8.8)	6 (15.4)	2 (3.9)		2 (6.5)	6 (10.0)	
Dizziness	4 (4.4)	2 (5.1)	2 (3.9)		0 (0.0)	4 (6.7)	
Tinnitus	3 (3.3)	3 (7.7)	0 (0.0)		1 (3.2)	2 (3.3)	
Hypoacusia	9 (9.9)	8 (20.5)	1 (1.9)		4 (12.9)	5 (8.3)	
Ophthalmological	35 (38.5)	25 (64.1)	10 (19.2)	**< 0.001**^**d**^	20 (64.5)	15 (25.0)	**< 0.001**^**d**^
Cornea verticillata	27 (29.7)	24 (61.5)	3 (5.8)		18 (58.1)	9 (15.0)	
Retinal vasculopathy	5 (5.5)	4 (10.3)	1 (1.9)		3 (9.7)	2 (3.3)	
Cataracts	6 (6.6)	0 (0.0)	6 (11.5)		1 (3.2)	5 (8.3)	
Dry eyes	3 (3.3)	1 (2.6)	2 (3.9)		2 (6.5)	1 (1.7)	
Cardiac	45 (49.5)	24 (61.5)	21 (40.4)	**0.046**^**d**^	23 (74.2)	22 (36.7)	**0.001**^**d**^
LVH	35 (38.5)	19 (48.7)	16 (30.8)		20 (64.5)	15 (25.0)	
Dyspnoea	10 (11.0)	7 (18.0)	3 (5.8)		4 (12.9)	6 (10.0)	
Reduced exercise tolerance	9 (9.9)	6 (15.4)	3 (5.8)		2 (6.5)	7 (11.7)	
Syncope	5 (5.5)	5 (12.8)	0 (0.0)		1 (3.2)	4 (6.7)	
Gadolinium retention on MRI^b^	2 (2.2)	1 (2.6)	1 (1.9)		1 (3.2)	1 (1.7)	
Heart failure	8 (8.8)	6 (15.4)	2 (3.9)		4 (12.9)	4 (6.7)	
Bradycardia	5 (5.5)	4 (10.3)	1 (1.9)		2 (6.5)	3 (5.0)	
Auricular fibrillation	2 (2.2)	1 (2.6)	1 (1.9)		1 (3.2)	1 (1.7)	
Ventricular tachycardia	4 (4.4)	2 (5.1)	2 (3.9)		1 (3.2)	3 (5.0)	
Sudden cardiac death	1 (1.1)	1 (2.6)	0 (0.0)		0 (0.0)	1 (1.7)	
Gastrointestinal	13 (14.3)	7 (18.0)	6 (11.5)	0.387^d^	7 (22.6)	6 (10.0)	0.122^e^
Dermatological (i.e., angiokeratoma)	11 (12.1)	7 (18.0)	4 (7.7)	0.195^d^	7 (22.6)	4 (6.7)	**0.041**^e^
Renal	18 (19.8)	8 (20.5)	10 (19.2)	0.879^d^	7 (22.6)	11 (18.3)	0.630^d^
Albuminuria	5 (5.5)	3 (7.7)	2 (3.9)		1 (3.2)	4 (6.7)	
Proteinuria	10 (11.0)	5 (12.8)	5 (9.6)		5 (16.1)	5 (8.3)	
Decreased GFR	7 (7.7)	2 (5.1)	5 (9.6)		2 (6.5)	5 (8.3)	
Dialysis	2 (2.2)	1 (2.6)	1 (1.9)		1 (3.2)	1 (1.7)	
Kidney transplant	1 (1.1)	1 (2.6)	0 (0.0)		1 (3.2)	0 (0.0)	
Cerebrovascular	10 (11.0)	8 (20.5)	2 (3.9)	**0.017**^e^	7 (22.6)	3 (5.0)	**0.028**^e^
White matter lesion on MRI^c^	2 (2.2)	2 (5.1)	0 (0.0)		2 (6.5)	0 (0.0)	
TIA	2 (2.2)	2 (5.1)	0 (0.0)		1 (3.2)	1 (1.7)	
Stroke	3 (3.3)	2 (5.1)	1 (1.9)		2 (6.5)	1 (1.7)	

Multiple answer, the sum of percentages can be greater than 100%

GFR, glomerular filtration rate; GI, gastrointestinal; LVH, left ventricular hypertrophy; Lyso-Gb3, plasma globotriaosylsphingosine; MRI, magnetic resonance imaging; NA, not available; PNS, peripheral nervous system; TIA, transient ischemic attack

^a^Any signs included: PNS, GI, opththalmological, cardiac, respiratory, dermatological, vascular, cerebrovascular, lymphatics, renal, neuropsychological, bones and elevated Lyso-Gb3; ^b^Patients with ≥ 1 cardiac MRI performed n = 6; ^c^Patients with ≥ 1 brain MRI performed n = 3; ^c^X^2^-Square; ^d^Fisher's test

Statistically significant *p* values (*p* <0.05) are highlighted in bold

Focusing on the 56.7% of untreated females with major organ involvement (n = 34), both mean age (and age distribution) and presence of comorbidity, were comparable to treated patients, (*P* = 0.8 and *P* = 0.8, respectively, data not shown). A detailed description of these patients is provided in Additional file 2: Table S2 and Additional file 3: Table S3: Both classic and non-classic phenotypes were present. Eighteen females in Additional file 2: Table S2 had major organ involvement together with a typical Fabry sign, with 13 presenting either cardiovascular, renal, or cerebrovascular involvement, while the other 5 had GI or PNS involvement. The remaining 16 cases in Additional file 3: Table S3 had major organ involvement, but no typical sign of FD, and 14 had at least cardiovascular or renal involvement. In total, 27 females in the untreated subgroup presented at least cardiovascular, renal, or cerebrovascular involvement, organs highlighted in European recommendations for potential specific treatment initiation.

Of note, for those patients who received treatment in this cohort, cardiac disease (12/31; 38.7%) was the most frequent factor leading to treatment initiation (Table 4). Nevertheless, 22 females out of the 60 untreated had cardiac involvement (Table 3).

**Table 4:** Factors leading to treatment initiation (females with VUS excluded)

Factors	N = 31 n (%)
Neuropathic pain	6 (19.4)
Proteinuria	1 (3.2)
GFR decreased	1 (3.2)
Stroke	2 (6.5)
TIA	1 (3.2)
Cardiac disease	12 (38.7)
GI symptoms	1 (3.2)
Exercise intolerance and hypohidrosis	2 (6.5)
Lyso-Gb3 increased	1 (3.2)
Angiokeratoma	3 (9.7)
Other^a^	17 (54.8)

Multiple answer, the sum of percentages can be greater than 100%

α-Gal-A, alpha galactosidase A; TIA, transient ischemic attack

^a^Mainly: *GLA* variant detection (n = 3), confirmatory renal biopsy (n = 3) and acroparesthesias (n = 2); other factors selected by physicians included access to the drug, decreased α-Gal A, presence of cornea verticillata, chronic venous insufficiency or microalbuminuria

### Clinical events

Clinical events occurred in 11 (11.3%) patients within the follow-up period (between diagnosis and last assessment), mainly cerebrovascular, (4 strokes, 1 transient ischaemic attack [TIA] and 1 new white matter lesion [WML] identification), followed by cardiac (1 arrythmia, 1 congestive heart failure and 2 cardiac procedures (1 vascular shunt and 1 not available), and 1 renal (change in estimated glomerular filtration rate [eGFR] category accompanied by ≥ 25% decline). At the time of analysis, 3 (3.1%) patients had died (1 from pancreatitis, 2 from heart failure).

## Discussion

This study describes the clinical characteristics of a Spanish cohort of 97 females with an identified *GLA* variant. We found that although the median age for the entire cohort at study inclusion was 50 years, it was only about a median time of 3 years earlier (36.1 months) when they were identified with a *GLA* variant. This suggests that efforts to increase diagnosis in the female population are being made in recent years in Spain. As FD is a progressive multisystemic disease with a reduced life expectancy, early diagnosis is essential. Other authors have found pedigree analysis is the most effective strategy for diagnosis of FD in females with affected family members [20, 28]. Our results support this, as most females with a *GLA* variant in this cohort were identified by family screening (70%). Six out of 97 presented with a VUS. Regarding the remaining 91 females, variants associated with classic and non-classic phenotypes were similarly distributed (40% and 54%, respectively), which is in line with the literature [29, 30]. Missense variants were identified in 84 (87%) females, of which 72% of the variants fell within the classic type, demonstrating that carrying a missense variant does not exclude this associated phenotype. Overall, females with classic variants in this cohort had a higher burden of disease. However, given the rarity of the disease, the almost 1000 known *GLA* variants and the variation in clinical expressivity, determining genotype–phenotype associations in FD is complicated. Consequently, phenotype classification is currently lacking for many *GLA* variants [31]. The evolving understanding of the natural course of the disease suggests that it is more appropriate to describe FD as a disease with a broad spectrum of heterogeneously progressive clinical phenotypes. Indeed, females in the non-classic subgroup also had a high prevalence of disease signs and symptoms, with major organ involvement (n = 32, 62%). Overall, these results suggest that females with pathogenic variants of FD, regardless of the type of variant, will likely develop disease symptoms in a high percentage of cases.

The most common manifestation of FD in our cohort was cardiac (50%), with LVH occurring in 39%, followed by ophthalmological (39%), PNS (35%) and renal (20%) manifestations. When this cohort was compared to other cohorts from Spain and other countries [25, 26], overall rates of cardiac disease or LVH were very much aligned when similar age ranges are considered. PNS or renal involvement was sometimes higher probably due to differences in age and phenotype of the subjects included [25, 26].

Regarding specific treatment of adult patients with FD, it should be directed to prevention of further progression to irreversible tissue damage and organ failure. The current therapeutic options for FD include ERT and migalastat for patients with amenable gene mutations, along with supportive care to manage symptoms [21, 22, 32]. As mentioned before, family screening is not only key for early diagnosis but also to enable timely treatment [17, 21, 22]. This is shown in our results, as 55% of the 33 females who were receiving disease-specific therapy were diagnosed by family screening. In contrast to this, it is striking that 48% of patients identified by clinical suspicion did not receive specific treatment.

The initiation of specific therapy in females requires demonstration of signs or symptoms attributable to FD disease, in addition to carrying a disease-causing mutation in the *GLA* gene [33, 34].

Overall, the proportion of patients with signs or symptoms of FD was significantly higher among treated patients, where the vast majority presented with major organ involvement and a typical Fabry sign, and mostly associated with a classic type of variant. In contrast, a high percentage of females with a non-classic variant remained untreated. This finding points to type of variant as influencing treatment initiation. From a clinical perspective, it is not surprising that cardiac disease was named as the leading reason for treatment, since it was the most prevalent manifestation overall in this cohort. Indeed, cardiac involvement was also the most prevalent manifestation among the untreated females in this cohort. In the German cohort of Lenders et al. 2016 [25], the presence of LVH also had a differential impact on treatment initiation compared to renal involvement, which was also prevalent but mild.

In total, we found that 34 females were not receiving specific treatment despite presenting at least one major organ involvement, and age or comorbidities, which did not differ overall from those treated. Age and comorbidities showed no significant differences when compared to the treated population. At an individual level, there might be several reasons for females not being treated: a diagnosis of FD was recently made so treatment initiation was only possible after patient inclusion in the study, presence of comorbidities might make it difficult to attribute signs or symptoms to FD, advanced age might be a limitation depending on individual characteristics and treatment expectations, rejection of specific treatment by the patient, and access barriers.

Close follow-up and thorough assessment of Fabry females is encouraged, to be able to confirm or discard whether signs and symptoms are FD related. LVH is considered as the main hallmark of FD cardiac involvement. However, there is growing evidence that females especially may develop myocardial fibrosis without detectable LVH [35, 36]. Indeed, in this cohort, females having cardiac MRI evaluation were very few, as well as those having brain MRI. Other organ involvement, such as gastrointestinal or other PNS damage may be underestimated due to the lack of objective monitoring tools.

We acknowledge our study has several limitations. First, it is a retrospective and multicentre study which can influence data collection. Second, although site selection tried to represent the actual diversity of FD females in the country, a bias in site selection cannot be avoided due to the impossibility to consider the inclusion of all existing females with *GLA* variants in the country. Third, the study of rare diseases is inherently hampered by their low prevalence, which makes it difficult to obtain a complete picture of clinical involvement and treatment. Fourth, influence of comorbidities unrelated to FD cannot be excluded.

Nevertheless, our study contributes to a better knowledge of the disease spectrum in a cohort of Spanish Fabry females and its associated management: it is the biggest Spanish female cohort investigated to date, with certain parameters being explored for the first time, such as plasma lyso-Gb3, α-Gal A activity or the influence of age, comorbidities, or type of variant associated phenotype in specific treatment initiation. It also describes the reason for diagnosis (clinical suspicion or family screening) and explores its alignment with current European recommendations for treatment initiation.

In conclusion, the findings in this study highlight the increasing efforts made in clinical practice in recent years in the diagnosis of Fabry females and in their timely treatment by performing family screening. A high percentage of females with a *GLA* pathogenic variant will likely develop symptoms attributable to the disease, regardless of their associated phenotype. A fraction of females with severe disease in this cohort is receiving specific treatment. Nonetheless, a significant number of females, even with the same profile as the ones treated, and who may be eligible for treatment according to European recommendations, remained untreated. Reasons for this merit further investigation.

## Methods

### Study design and participants

This was a multicentre, retrospective medical charts review study on FD conducted at 12 Spanish hospitals, in accordance with the Declaration of Helsinki and national regulations. Study investigators represented different specialties (cardiology, nephrology, and internal medicine). The study was approved by the ethics committees of all participating centres, and written informed consent was obtained from all alive patients.

Eligible patients were females aged ≥ 16 years with a confirmed *GLA* variant.

The primary objective was to retrospectively describe a Spanish cohort of females with a variant in the *GLA* gene. Secondary objectives included determining clinical differences in subgroups associated with several characteristics, such as age, type of gene variant, α-Gal A activity and plasma lyso-Gb3 levels. Regarding type of gene variant, the associated phenotype was assigned based on the public databases with this information [31, 37, 38]. For variants not included in these references or with discrepant classification, the associated phenotype was given based on the investigator’s experience. Also, as a secondary endpoint, differences by specific treatment initiation or not were analysed, as well as the factors leading to treatment initiation.

Patients’ information was retrieved from their medical charts, including demographics, genetics, family and personal medical history, laboratory tests, comorbidities, as well as survival status and last follow-up.

### Statistical considerations

A sample size of 71 females was estimated as realistic to be enrolled, considering a FD prevalence of 1/65,000, as an intermediate value within the range given in the literature (40,000 [0.0025%] [5] to 117,000 [0.0009%]) [6], which is 684 FD patients out of a Spanish population of 44.5 M. We then considered a 50:50 distribution of males and females in the Spanish population (342 females), the exclusion of children in the study, and the number of female cases published in the FOS registry, as a reference (n = 49) [27]. For a given characteristic that appears in at least 15% of the studied population, the random inclusion of 71 patients will yield a precision of estimations of ± 7.67% for a two-sided analysis, with an alpha error of 0.05, assuming a maximum of data loss of up to 5% of cases (due to incomplete, inconsistent, or non-processable information for any other reason).

Categorical variables were described as frequencies and percentages and continuous variables as means ± standard deviation, medians, and ranges. Data collected from medical records included comparisons between subgroups that were done using the Mann–Whitney or t-test for continuous variables and the Chi-squared or Fisher’s exact test for binary variables, as appropriate.

For exploratory analyses done regarding “type of organ involvement”, major organ involvement was defined as cardiovascular, renal, cerebrovascular, PNS or gastrointestinal involvement. Typical Fabry signs were defined as angiokeratoma, cornea verticillata, or elevated plasma Lyso-Gb3.

We also evaluated clinical events including cardiac (i.e., myocardial infarction, arrhythmias, cardiac syncope, congestive heart failure, angina pectoris or need for cardiac procedures such as pacemaker, bypass, stenting, valve replacement or transplantation [39]), renal (i.e., incident urinary albumin creatinine ratio [UACR] ≥ 30 mg/g, worsening nephropathy: UACR > 300 mg/g, total protein loss of > 1000 mg/24 h, eGFR < 15 mL/min/1.73 m^2^; annual decrease in eGFR > 5 ml/min/1.73m^2^, change in eGFR category accompanied by ≥ 25% decline, kidney transplant, or dialysis [39, 40]), cerebrovascular (i.e., new WML identification by magnetic resonance imaging, TIA or strokes [39, 41]) and death during follow-up (between diagnosis and last assessment).

Missing data were not considered in the analyses and a significance level of 0.05 was used for statistical testing. The IBM SPSS Statistics version 22.0 (IBM Corp., Armonk, USA) was used for statistical analysis.

## Supplementary Information

**Additional file 1: Table S1.** Clinical description of patients with VUS

**Additional file 2: Table S2.** Clinical description of untreated patients with major organ involvement and typical Fabry signs

**Additional file 3: Table S3.** Clinical description of untreated patients with major organ involvement and without typical Fabry signs

## Data Availability

The datasets generated and/or analysed during the current study are not publicly available. Researchers may request data access, specifying their reasons for access to Amicus Therapeutics (aaguinaga@amicusrx.com). Request for access will be reviewed by the Amicus Therapeutics scientific committee. Additional results supporting the conclusions of this article are included in additional files.
