# Supplementary material for: The Spanish Fabry women study: a retrospective observational study describing the phenotype of females with GLA variants
Source: Orphanet J Rare Dis. 2023 Jan 9;18:8. doi: 10.1186/s13023-022-02599-w (PMC9830917; doi:10.1186/s13023-022-02599-w)
Supplement: Supplementary file 1 — Additional file 1: Table S1. Clinical description of patients with VUS [file 13023_2022_2599_MOESM1_ESM.docx]

Additional table 1. Clinical description of patients with VUS

| **Genetic variant** | **Treatment status** | **Major organ involvement** | | **Signs and symptoms** | **Typical signs of FD** | **Comorbidity** | **Age^a^ (years)** | **Time since *GLA* identification** |
| --- | --- | --- | --- | --- | --- | --- | --- | --- |
| R118C c.352C>T | Untreated | PNS | Acroparesthesias | | - | - | 29 | 4y 3m |
| R118C c.352C>T | Untreated | - | - | | - | - | 38 | 3y 5m |
| R118C c.352C>T | Treated | Cardiac, renal, GI, CV, PNS | LVH, pain, vertigo, dizziness, diarrhoea, constipation, white matter lesion on MRI, TIA, albuminuria, anxiety | | - | Carpal tunnel syndrome, obesity | 48 | 4y 1m |
| A143T c.427G>A | Untreated | - | Cataract | | Angiokeratoma | AHT | 72 | 1y 9m |
| A143T c.427G>A | Untreated | Cardiac | LVH, cardiac failure, atrial fibrillation, cataract, dyspnoea, RET | | - | Hypothyroidism | 63 | 2m |
| S126G c.376A>G | Treated | CV, PNS | TIA, acroparesthesias, depression, anxiety | | - | Heterozygosity for the factor V Leiden, AHT | 49 | 4y 8m |
| ^a^At study inclusion  Abbreviations: AHT, arterial hypertension; CV, cerebrovascular; FD, Fabry disease; GI, gastrointestinal; m, month; MRI, magnetic resonance imaging; PNS, peripheral nervous system; RET, reduced exercise tolerance; TIA, transient ischemic attack; y, year. | | | | | | | | |
