# Supplementary material for: The Spanish Fabry women study: a retrospective observational study describing the phenotype of females with GLA variants
Source: Orphanet J Rare Dis. 2023 Jan 9;18:8. doi: 10.1186/s13023-022-02599-w (PMC9830917; doi:10.1186/s13023-022-02599-w)
Supplement: Supplementary file 2 — Additional file 2: Table S2. Clinical description of untreated patients with major organ involvement and typical Fabry signs [file 13023_2022_2599_MOESM2_ESM.docx]

Additional table 2. Clinical description of untreated with major organ involvement and typical Fabry signs

| **Major organ involvement** | **Signs and symptoms** | **Typical signs of FD** | **Comorbidity** | **Age^a^ (years)** | **Time since *GLA* identification** | **Associated phenotype** |
| --- | --- | --- | --- | --- | --- | --- |
| Cardiac | LVH | Lyso-Gb3 | - | 62 | 5y 11m | Non-classic |
| Cardiac | LVH, eye dryness | Lyso-Gb3 | AHT | 75 | 3y 8m | Non-classic |
| Cardiac, renal, CV, PNS | LVH, dyspnoea, RET, bradycardia, TIA, albuminuria, proteinuria, GFR <90, hypoacusis, dizziness, pain | Cornea verticillata | Diabetes, AHT | 81 | 9y 8m | Classic |
| Cardiac, CV, and PNS | Cardiac failure, RET, LVH, dyspnoea, stroke, pain, hypoacusis, dizziness, CVD | Cornea verticillata | Diabetes, AHT, dyslipidaemia | 65 | 9y 7m | Classic |
| Cardiac, GI | Dyspnoea, wheezing, nausea, vomiting | Lyso-Gb3 | - | 57 | 2y 11m | Non-classic |
| Cardiac, GI | LVH, nausea, vomiting, cataract | Lyso-Gb3 | AHT, SLE | 67 | 6m | Non-classic |
| Cardiac, renal | LVH, albuminuria | Angiokeratoma | AHT | 53 | 2y 9m | Non-classic |
| Cardiac, renal, PNS | Acroparesthesia, syncope, albuminuria | Cornea verticillata, lyso-Gb3 | - | 39 | 6y 11m | Classic |
| Cardiac, GI, PNS | Hypoacusis, dizziness, nausea, vomiting, syncope | Lyso-Gb3 | - | 36 | 5y 7m | Classic |
| Cardiac, PNS | LVH, acroparesthesia, cataract | Lyso-Gb3 | Diabetes | 73 | 4y 6m | Non-classic |
| Cardiac, PNS | LVH, hypoacusis, cataract | Lyso-Gb3 | Diabetes, AHT | 84 | 2y 10m | Non-classic |
| Cardiac, PNS | LVH, pain crises | Angiokeratoma | - | 41 | 5m | Classic |
| Renal | GFR <90 | Lyso-Gb3 | Fibromyalgia | 71 | 1y 11m | Non-classic |
| GI | Diarrhoea | Lyso-Gb3 | Asthma | 42 | 2y | Non-classic |
| PNS | Dizziness | Angiokeratoma | Arterial insufficiency, basilar artery | 56 | 1y 8m | Non-classic |
| PNS | Dizziness, vertigo, dyspnoea, wheezing | Lyso-Gb3 | - | 48 | 2y 9m | Non-classic |
| PNS | Pain | Lyso-Gb3 | Diabetes, mild KF | 78 | 8y 7m | Non-classic |
| PNS | Acroparesthesia, pain | Cornea verticillata | - | 42 | 9y 7m | Classic |
| ^a^At study inclusion  Abbreviations: AHT, arterial hypertension; CV, cerebrovascular; CVD, conjunctival vascular disease; FD, Fabry disease; GFR. Glomerular filtration rate; GI, gastrointestinal; GLE, gadolinium late enhancement; KF, kidney failure; Lyso-Gb3, plasma globotriaosylsphingosine; LVH, left ventricular hypertrophy; month; PNS, peripheral nervous system; PVI, peripheral venous insufficiency; RET, reduced exercise tolerance; SAHS, sleep apnoea/hypopnoea syndrome; SLE, systemic lupus erythematosus; TIA, transient ischemic attack; y, year. | | | | | | |
