# Supplementary material for: The Spanish Fabry women study: a retrospective observational study describing the phenotype of females with GLA variants
Source: Orphanet J Rare Dis. 2023 Jan 9;18:8. doi: 10.1186/s13023-022-02599-w (PMC9830917; doi:10.1186/s13023-022-02599-w)
Supplement: Supplementary file 3 — Additional file 3: Table S3. Clinical description of untreated patients with major organ involvement and without typical Fabry signs [file 13023_2022_2599_MOESM3_ESM.docx]

Additional file 3. Clinical description of untreated patients with major organ involvement and without typical Fabry signs

| **Major organ involvement** | **Signs and symptoms** | **Comorbidity** | **Age^a^ (years)** | **Time since *GLA* identification** | **Associated phenotype** |
| --- | --- | --- | --- | --- | --- |
| Cardiac | Death (LVH, dyspnoea, syncope, cardiac failure, atrial fibrillation, and bradycardia) | - | 61 | NA | Classic |
| Cardiac | Atrial fibrillation | Irritable colon | 30 | 1y 8m | Non-classic |
| Cardiac | LVH, cataract | AHT, mild KF, SAHS, depression, claustrophobia | 63 | 1y 6m | Non-classic |
| Cardiac, GI | RET, nausea, vomiting | - | 23 | 2y 9m | Non-classic |
| Cardiac, renal | Death (cardiac failure, proteinuria, dialysis) | Diabetes, AHT, iron-deficiency, anaemia | 71 | 2y 6m | Non-classic |
| Cardiac, renal | LVH, albuminuria | - | 69 | 2y 7m | Non-classic |
| Cardiac, PNS | LVH, dyspnoea, RET, pain, hot/cold intolerance, dizziness, hearing loss | Asthma | 77 | 1y 2m | Classic |
| Cardiac, PNS | LVH, RET, syncope, pain, dizziness, | Neurological or psychiatric problems, treatment refusal | 66 | 4y 7m | Classic |
| Cardiac, PNS | Tachycardia, acroparesthesia | Obesity | 21 | 2y 10m | Non-classic |
| Cardiac, renal, CV | LVH, LGE, dyspnoea, heart failure, RET, cataract, conjunctival vasculopathy, GFR <90, TIA | AHT, PVI | 79 | 2y 3m | Non-classic |
| Renal | GFR <90 | - | 42 | 2y 3m | Non-classic |
| Renal | GFR <90, proteinuria | - | 42 | 1y 6m | Non-classic |
| Renal | Proteinuria | AHT | 54 | 1y 6m | Non-classic |
| Renal, PNS | Albuminuria, acroparesthesia | Obesity | 19 | 7m | Non-classic |
| PNS | Acroparesthesia, pain | AHT, right hand tremors, breast lumps | 40 | 1y 6m | Non-classic |
| GI, and PNS | Dizziness, nausea | Hypercholesterolemia | 44 | 8m | Non-classic |
| ^a^At study inclusion  Abbreviations: AHT, arterial hypertension; GFR, glomerular filtration rate; GI, gastrointestinal; LVH, left ventricular hypertrophy; LGE, late gadolinium enhancement; m, month; NA, not available; PNS, peripheral nervous system; RET, reduced exercise tolerance; y, year. | | | | | |
